# Living and dying to be counted: What we know about the epidemiology of the global adolescent HIV epidemic

**DOI:** 10.7448/IAS.20.4.21520

**Published:** 2017-05-16

**Authors:** Amy L. Slogrove, Mary Mahy, Alice Armstrong, Mary‐Ann Davies

**Affiliations:** ^1^ Centre for Infectious Disease Epidemiology and Research, School of Public Health and Family Medicine, Faculty of Health Sciences, University of Cape Town, Cape Town, South Africa; ^2^ Strategic Information and Monitoring Division, UNAIDS, Geneva, Switzerland; ^3^ Independent Consultant, London, United Kingdom

**Keywords:** Adolescent, HIV, transition, healthcare transition, transition to adulthood, surveillance, monitoring, UNAIDS, sub‐Saharan Africa

## Abstract

**Introduction**: With increasing survival of vertically HIV‐infected children and ongoing new horizontal HIV infections, the population of adolescents (age 10–19 years) living with HIV is increasing. This review aims to describe the epidemiology of the adolescent HIV epidemic and the ability of national monitoring systems to measure outcomes in HIV‐infected adolescents through the adolescent transition to adulthood.

**Methods**: Differences in global trends between younger (age 10–14 years) and older (age 15–19 years) adolescents in key epidemic indicators are interrogated using 2016 UNAIDS estimates. National population‐based survey data in the 15 highest adolescent HIV burden countries are evaluated and examples of national case‐based surveillance systems described. Finally, we consider the potential impact of adolescent‐specific recommendations in the 2016 WHO Consolidated Guidelines on the Use of Antiretroviral Drugs for Treating and Preventing HIV Infection.

**Discussion**: UNAIDS estimates indicate the population of adolescents living with HIV is increasing, new HIV infections in older adolescents are declining, and while AIDS‐related deaths are beginning to decline in younger adolescents, they are still increasing in older adolescents. National population‐based surveys provide valuable estimates of HIV prevalence in older adolescents and recent surveys include data on younger adolescents. Only a few countries have nationwide electronic case‐based HIV surveillance, with the ability to provide population‐level data on key HIV outcomes in the diagnosed population living with HIV. However, in the 15 highest adolescent HIV burden countries, there are no systems tracking adolescent transition to adulthood or healthcare transition. The strength of the 2016 WHO adolescent‐specific recommendations on antiretroviral therapy and provision of HIV services to adolescents was hampered by the lack of evidence specific to this age group.

**Conclusions**: Progress is being made in national surveillance and global monitoring systems to specifically identify trends in adolescents living with HIV. However, HIV programmes responsive to the evolving HIV prevention and treatment needs of adolescents can be facilitated further by: data disaggregation to younger and older adolescents and mode of HIV infection where feasible; implementation of tools to achieve expanded national case‐based surveillance; streamlining consent/assent procedures in younger adolescents and consensus on indicators of adolescent healthcare transition and transition to adulthood.

## Introduction

Globally in 2015, there were 1.2 billion adolescents aged 10–19 years (as defined by the World Health Organization), accounting for 16% of the world's population [1–3]. Adolescence is characterized by unique and rapid biological and psychosocial changes with evolving capacities during the transition from childhood to adulthood [3]. Almost 1.8 million adolescents are living with HIV, 80% of whom live in sub‐Saharan Africa [4]. The adolescent population living with HIV comprises vertically (perinatally and postnatally) and horizontally (behaviourally) HIV‐infected adolescents. With increasing survival of vertically HIV‐infected children into adolescence and ongoing new horizontal HIV infections, this population is growing [5,6].

Compared to HIV‐infected children and adults, HIV‐infected adolescents have poorer retention in care, lower rates of virologic suppression and higher rates of mortality [7–9]. Different risks and barriers experienced during the stages of younger (age 10–14 years) and older (age 15–19 years) adolescence may impact on these HIV‐related outcomes as well as on the risk for HIV acquisition. Younger adolescents are more often living at home, are in early stages of puberty and fewer have reached sexual debut compared with older adolescents. During older adolescence, gender differences in HIV acquisition risk become apparent with risk higher among older adolescent girls than boys and additional vulnerabilities arise for adolescent key populations (young men who have sex with men, transgender people, young sex workers and those who inject drugs) [10]. Since all‐cause mortality rates in the general adolescent population are lower than in other age groups, adolescents have been regarded as a healthy population not requiring much attention of healthcare services [11,12]. Consequently, national health information systems have not been oriented towards monitoring adolescent health. Global HIV monitoring systems have until recently ignored this vulnerable age group by aggregating adolescents aged 10–14 years with all children under 15 years of age and those aged 15–19 years with all people 15 years and older. This has posed a challenge for monitoring adolescents as a unique target population as well as understanding the differences between younger and older adolescents.

The adolescent developmental transition to adulthood coincides in some settings with a healthcare transition from paediatric to adult HIV services. There is concern that outcomes following this healthcare transition are worse than in paediatric HIV services prior to the transition [13–15]. However, in much of sub‐Saharan Africa, where HIV care is implemented at primary healthcare clinics, there is no separation in paediatric and adult services and thus no need for transition of care [16]. Nevertheless, transition to adulthood still presents challenges for optimal HIV disease management as adolescent health needs evolve and autonomy for their own self care is necessary [17]. Many of these challenges are shared by both vertically and horizontally infected adolescents, although their disease progression and severity during adolescence may differ [18,19]. To better understand the reasons for poorer outcomes in adolescents compared to other age groups, and the contributions of the transition to adulthood as well as the potential impact of healthcare transition, consensus is needed across settings for both research and surveillance on how to recognize and define when healthcare transition has occurred and ultimately how to identify successful transition through adolescence to adulthood.

## Methods

The most recent estimates and trends in the size of the global adolescent HIV epidemic are reviewed. We interrogate global and country‐specific trend differences (for the five highest adolescent HIV burden countries) between younger and older adolescents in the total number living with HIV, the number of AIDS‐related deaths and the number of new infections using the 2016 UNAIDS estimates for 1990–2015. Estimates from 1990 through 2015 are used and are based on the most recent 2016 UNAIDS Spectrum model [20,21] for the current and historical years. Recent, nationally representative data from periodic population‐based surveys in the 15 highest adolescent HIV burden countries are evaluated and estimates of adolescent HIV prevalence presented where available. Three examples of continuous national case‐based surveillance systems (United Kingdom/Ireland, Brazil and South Africa) and their capacity to track younger and older adolescent outcomes are described. Finally, the potential impact is considered of the three new adolescent‐specific recommendations in the 2016 World Health Organization (WHO) Consolidated Guidelines on the Use of Antiretroviral Drugs for Treating and Preventing HIV Infection [22].

## Discussion

### Trends in the global adolescent HIV epidemic

#### UNAIDS supported estimates

Annually UNAIDS publishes global, regional and national estimates of key HIV epidemic indicators including age‐ and sex‐specific estimates of the number of people living with HIV, the number of people newly infected with HIV and the number of people dying from AIDS‐related causes [20]. As it is currently not possible to directly measure these indicators in most countries, UNAIDS assists countries annually to develop modelled estimates of indicators that are informed by demographic, HIV epidemiologic and programmatic data [20]. The generation of country‐specific estimates is facilitated by the Spectrum software used by countries to input their available country‐specific data into the epidemic model and develop national estimates. Spectrum estimates are reviewed by UNAIDS for validity and consistency and are then compiled to develop regional and global estimates. Two important improvements were made to the 2016 UNAIDS estimates model specific to understanding the paediatric and adolescent HIV epidemics [21]. Based on an updated review (produced by Lynne Mofenson and commissioned by UNAIDS, and detailed in Annex 2 of reference [21]), on the probability of HIV transmission during pregnancy, delivery and breastfeeding, the number of children estimated to have become HIV infected was revised downward for current and historical years. Based on multiregional analyses performed by the International Epidemiologic Database to Evaluate AIDS (IeDEA), improvements were also made to more accurately reflect at what age children initiate ART [23,24]. Rather than assuming that a constant proportion of children less than age 15 years initiate ART as they become eligible as done in previous models, the current model uses age‐specific assumptions of the proportion of children starting on ART [3,20]. This resulted in a downward correction in the estimated number of surviving children in the previous years and thus also a downward correction in the number of adolescents living with HIV.

**Figure 1:**
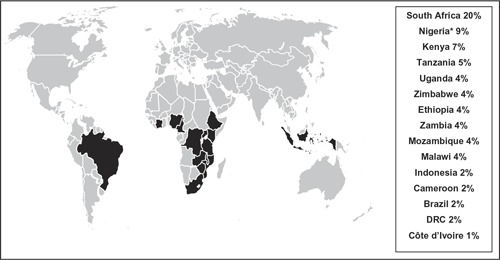
**Global distribution of HIV‐infected adolescents (age 10**–**19 years) in 2015, according to 2016 UNAIDS estimates, illustrating the 15 highest burden countries** [4]. * Nigeria unofficial preliminary 2016 results; DRC – Democratic Republic of Congo

It is estimated that 50% of HIV‐infected adolescents live in just six countries and that 15 countries account for 75% of all HIV‐infected adolescents globally, 13 countries in sub‐Saharan Africa, one in Asia and one in South America [4] (Figure 1). In 2015, there were approximately 1.76 million (uncertainty bound (UB) 1.47 million‐2.19 million) adolescents living with HIV, 36% aged 10–14 years and 64% aged 15–19 years [4]. Although this estimated number of adolescents living with HIV has been revised downward compared to 2014 estimates of approximately 2.0 million (UB 1.9–2.3 million), it is important to recognize that this correction is due to a reduced estimated number of new child infections since the start of the epidemic and improved assumptions around ART initiation that result in higher AIDS‐related mortality rates during the childhood years; and is not necessarily due to a change in outcomes of adolescents living with HIV [4,20].

**Figure 2:**
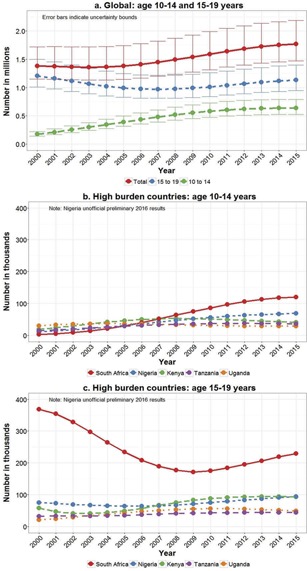
Number of adolescents living with HIV according to 2016 UNAIDS estimates (2000–2015).

**Figure 3:**
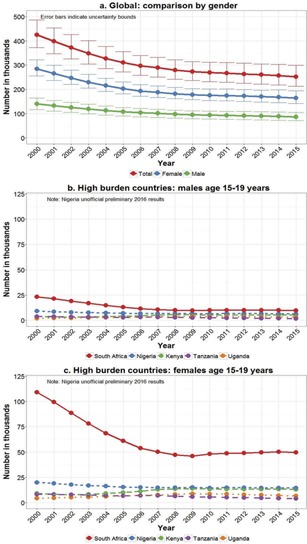
New HIV infections in people age 15–19 years according to 2016 UNAIDS estimates (2000–2015).

During the last decade, with expansion of paediatric antiretroviral therapy (ART) and its survival benefits, in combination with approximately 250,000 (UB 220,000–330,000) new infections in older adolescents annually, the number of adolescents living with HIV has risen 28% between 2005 and 2015 (Figure 2a). Sixty‐five per cent of new infections are in older adolescent girls, with this gender discrepancy greatest in Eastern and Southern Africa where 76% of estimated new adolescent infections in 2015 were in girls aged 15–19 years. Some of the high‐burden countries are seeing reductions in new older adolescent infections; however, these numbers are still worryingly high (Figure 3b and 3c). In South Africa, for example, new infections in older adolescents peaked in 1999 at 120,000 (UB 110,000–130,000) new infections in girls and 24,000 (UB 19,000–30,000) new infections in boys. In 2015, this had more than halved to 49,000 (UB 43,000–57,000) new infections in older adolescent South African girls and 9,800 (UB 7,400–12,000) in older adolescent boys. These estimates are consistent with trends observed in consecutive South African National HIV Surveys between 2002 and 2012 [25]. The large decline between 2002 and 2009 in South African older adolescents living with HIV, seen in Figure 2c, may partly be explained by declining numbers of new infections in this age group in combination with poor survival of vertically HIV‐infected children prior to national expansion of ART services.

**Figure 4:**
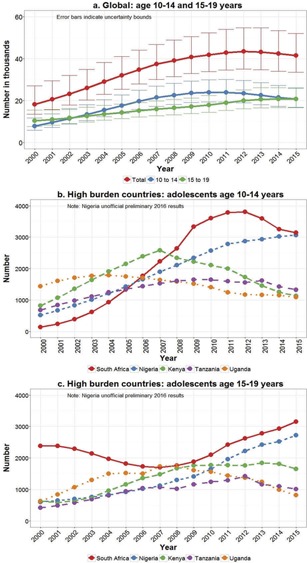
Adolescent AIDS‐related deaths according to 2016 UNAIDS estimates (2000–2015).

AIDS‐related deaths in all adolescents 10–19 years of age peaked in 2012 at approximately 43,900 (UB 35,000–55,000) deaths globally and have since seen a small decline each year to 40,800 (UB 33,500–52,000) deaths in 2015 (Figure 4a). This pattern is quite different though when stratified by younger and older adolescents. Annual deaths in younger adolescents peaked in 2010 at approximately 24,000 (UB 19,300–30,000) and then declined 16% to approximately 20,000 (UB 16,700–26,000) deaths in 2015. Deaths in older adolescents however have continued to rise each year with an estimated 20,800 (UB 16,800–26,000) deaths in 2015, and now equivalent numbers of deaths in younger and older adolescents (Figure 4a). Amongst the five countries with the highest burden of adolescent HIV – South Africa, Nigeria, Kenya, Tanzania and Uganda – only Nigeria is still experiencing an increase in young adolescent deaths (Figure 4b). In Kenya, young adolescent deaths have halved since peaking in 2007 and South Africa has seen a reduction of 17% since 2012. Older adolescent deaths continue to increase though particularly in South Africa and Nigeria (Figure 4c).

The increasing AIDS related deaths among adolescents between 2005 and 2013 are potentially a reflection of the “bulge” of vertically HIV‐infected children aging into the adolescent age group. Following expansion of ART services, particularly in sub‐Saharan Africa, between 2005 and 2015 over 100,000 additional children living with HIV aged into adolescence each year, as the “bulge” of children vertically infected 10 years earlier (between 1995 and 2005) survived into adolescence. The number of children living with HIV that aged into adolescence increased from 53,000 in 2000 to 100,000 in 2005. Thus the proportion of adolescents living with HIV and at risk of AIDS deaths increased. The number of adolescents living with HIV entering adolescence is starting to decline, reflecting the increase in vertical HIV transmission prevention services from 2005 until 2015 and the dramatic decline in children newly infected with HIV 10 years earlier.

It is assumed that deaths in older adolescents are predominantly among vertically and not newly horizontally infected adolescents since horizontally infected adolescents will only recently have been infected; however, we are not aware of direct data to confirm this. As more children are reached with ART in future years, the adolescents living with HIV who were vertically infected will live longer. However, the contribution of this increased longevity to the total number of adolescents living with HIV will be offset by the rapid decline in the entrants to the adolescent HIV population as the number of children infected vertically between 2005 and 2015 has declined [4,26]. Thus, the number of adolescent AIDS‐related deaths is expected to decline while the mortality rate among the declining numbers of vertically infected adolescents needs to be monitored carefully to see that it does not increase. Among the adult population ≥20 years, the growing population of vertically infected young adults will be quantitatively overwhelmed by the much larger numbers of horizontally infected young adults. Understanding differences and intervening on the risk of mortality in vertically compared to horizontally HIV‐infected older adolescents and young adults is difficult unless these two groups can be differentiated in surveillance systems and in global estimates.

#### CIPHER mortality estimates

The Collaborative Initiative for Paediatric HIV Education and Research (CIPHER) pooled individual retrospective data from 12 cohort networks, representing 50 countries and five regions of the world [27]. Mortality in younger adolescents was estimated in 38,187 perinatally HIV‐infected adolescents, 79% from sub‐Saharan Africa. In perinatally HIV‐infected adolescents born between 1982 and 2005, the estimated cumulative incidence of mortality between 10 and 14 years of age was 2.6% (95% confidence interval (CI) 2.4–2.8%) [27]. However, since 11.3% (95% CI 10.9%‐11.8%) of adolescents were lost to follow‐up, this figure should be considered an underestimate of mortality. This is also likely to represent the best case scenario as the adolescents in these cohorts were all receiving HIV care and had all survived to at least 10 years of age in an era of limited treatment access. Further analysis on changes in younger adolescent mortality over time are expected from CIPHER in the near future.

Estimates indicate that HIV‐associated mortality is declining for younger vertically infected adolescents. Although this may seem positive, it is possible that the mortality burden is being displaced from younger to older adolescence as cohorts age up, with older adolescent mortality continuing to increase in the majority of high‐burden countries. Older adolescence coincides with the potential healthcare transition from paediatric to adult HIV services, as well as the most challenging developmental transitional stages on the road to adulthood. Disentangling the contribution of the healthcare transition and the transition to adulthood to rising mortality in older adolescents is going to require identification within surveillance systems of the younger and older adolescent age groups as well as indicators of adolescent transition to adulthood and, if relevant, the time of transfer between paediatric and adult HIV care services.

### National surveillance systems

#### Periodic national surveys

National surveys conducted periodically in countries with high HIV prevalence can be a valuable source for understanding the status of the HIV epidemic in individual countries. These are most often household surveys conducted in a nationally representative sample of the population and can include interviews as well as minimally invasive investigations such as blood pressure measurements and blood sampling for HIV. Due to challenges with obtaining consent for HIV testing in children and younger adolescents, as well as the large sample sizes required for robust estimates in these relatively low HIV‐prevalence age groups, until recently national surveys have seldom included people younger than 15 years of age. Table 1 summarizes the most recent available national population‐based surveys in the 15 adolescent high burden countries.

**Table 1:** Summary of adolescent HIV prevalence estimates from population‐based surveys in the 15 adolescent HIV high‐burden countries

			Age‐disaggregated prevalence (95% CI or N tested, given where reported)	
Country	% Global Adolescent HIV Burden	Year of Survey	Younger adolescents (age 10–14 years)	Older adolescents (age 15–19 years)
South Africa [25]	20%	2012		Total 3.2% (CI 2.4–4.1)
				Female 5.6% (CI 4.2–7.5)
				Male 0.7% (CI 0.4–1.2)
Nigeria [34]	9%^a^	2012		Total 2.9%
Kenya [32]	7%	2012	Total 0.6% (CI 0.2–0.9)	Total 1.0%
				Female 1.1% (CI 0.4–1.8)
				Male 0.9% (CI 0.1–1.8)
Tanzania [35]	5%	2011		Total 1% (*N* = 4097)
				Female 1.3% (*N* = 2153)
				Male 0.8% (*N* = 1944)
Uganda [36]	4%	2011		Total 2.4% (*N* = 2228)
				Female 3.0% (*N* = 2399)
				Male 1.7% (*N* = 2055)
Zimbabwe [30]^b^	4%	2015	Female 2.9% (CI 1.7–3.9)	Female 4.0% (CI 3.1–4.8)
			Male 2.4% (CI 1.5–3.0)	Male 3.5% (CI 2.4–4.3)
Ethiopia [37]	4%	2011		*15–49 years: Total 1.5%*
Zambia [29]^b^	4%	2016	Female 1.1% (CI 0.5–1.5)	Female 3.3% (CI 2.4–3.9)
			Male 0.8% (CI 0.2–1.2)	Male 1.8% (CI 1.0–2.3)
Mozambique [31]	4%	2009	10–11 years:	Total 5.0% (CI 3.6–6.4)
			Total 1.4% (CI 0.5–2.3)	Female 7.1% (CI 4.8–9.5)
			12–14 years:	Male 2.7% (CI 1.4–4.0)
			Total 1.8% (CI 1.0–2.8)Female 1.2% (CI 0.3–2.1)Male 2.5% (CI 1.1–3.9)	
Malawi [28]^b^	4%	2015	Female 1.9% (CI 0.8–2.7)	Female 2.0% (CI 1.1–2.7)
			Male 1.9% (CI 0.6–3.0)	Male 0.8% (CI 0.3–1.1)
Indonesia [33]	2%	2012	Not available	Not available
Cameroon [38]	2%	2011		Total 1.2% (*N* = 3198)
				Female 2.0% (*N* = 1647)
				Male 0.4% (*N* = 1551)
Brazil^b^ [39]	2%	2015	*Detection rate*	*Detection rate*
			*Female 0.7/100,000*	*Female 0.7/100,000*
			*Male 0.6/100,000*	*Male 4.2/100,000*
Democratic Republic of Congo [40]	2%	2013		Total 0.5% (*N* = 3713)
				Female 0.7% (*N* = 2021)
				Male 0.2% (*N* = 1692)
Côte d’Ivoire [41]	1%	2011		Total 0.5% (*N* = 1754)
				Female 0.8% (*N* = 965)
				Male 0.1% (*N* = 789)

^a^Based on unofficial preliminary 2016 UNAIDS estimates. ^b^Prevalence values deduced from graphs released in preliminary reports; CI – confidence interval.

With the recent release of preliminary results for Malawi, Zambia and Zimbabwe from the Population‐Based HIV Impact Assessments (PHIA) being conducted in the President's Emergency Plan for AIDS Relief (PEPFAR)‐supported countries, progress is being made through age disaggregation of the data to better understand HIV prevalence in adolescents and particularly the differences between younger and older adolescents [28–30]. Prior to this, only Kenya and Mozambique had population‐based estimates of prevalence in the 10–14 year age group [31,32]. The South African survey had the potential to provide an estimate for younger adolescents, having included a prevalence estimate in children aged 5–14 years [25]. The South African survey also refers to 12–14‐year olds as pre‐adolescents, highlighting the need for harmonization of the definition of adolescents [25]. The Indonesian Demographic and Health Survey (DHS) included questions on knowledge, attitudes and behaviours related to HIV, but due to the overall low prevalence of HIV in Indonesia and the epidemic being concentrated in key populations and not generalized in the population, did not include HIV testing and there is no population‐based HIV prevalence estimate in the Indonesian adolescent population [33].

Where available, HIV‐prevalence estimates in younger adolescents range from 0.6% (95% confidence interval (CI) 0.2–0.9) in Kenya to 2.9% (95% CI 1.7–3.9) in Zimbabwe, with little difference in this age group by gender [28–32]. In older adolescents, however, prevalence estimates reached 5% (95% CI 3.6–6.4) in Mozambique with a marked gender discrepancy [31], HIV prevalence being up to eight times higher in South African older adolescent females (5.6%, 95% CI 4.2–7.5) than males (0.7%, 95% CI 0.4–1.2) [25]. The Kenyan AIDS Indicator Survey observed a four times greater HIV prevalence in urban (2.2%) compared to rural (0.5%) older adolescents [32]. This is more than double the relative urban/rural difference of any other adult age group highlighting the vulnerability of urban adolescents in this setting. Due to their large general population sizes, Indonesia and Brazil are estimated to each account for 2% of the global population of adolescents living with HIV [4], although HIV prevalence in younger and older adolescents in Indonesia and Brazil is expected to be far lower than that seen in the sub‐Saharan African high burden countries, likely <0.1%. There is no population‐based HIV prevalence estimate for adolescents in Indonesia, and in Brazil which reports the HIV detection rate in younger and older adolescents, older adolescent males have a higher HIV detection rate than older adolescent females, in contrast to the pattern in sub‐Saharan Africa (Table 1).

#### Examples of national case‐based HIV programme surveillance

Complementary to surveys that provide detailed demographic, behaviour and other health‐related information on a sample of the population, national case‐based surveillance systems are essential to obtain reliable individual‐level HIV‐specific outcome data on the entire population living with HIV. These case‐based surveillance systems, when implemented nationally, can provide the absolute numbers of people diagnosed with HIV, the proportion that ever received ART, that remain on ART and that are virologically suppressed. Such a surveillance system can also provide data for specific geographic areas and more routinely than a household survey. In the absence of national case‐based data, estimation of outcomes using mathematical models is required. For the adolescent population living with HIV, case‐based national monitoring has the potential to be very useful and avoids relying on modelled estimates that due to the low prevalence of HIV in this age group are hampered by imprecision. Capacity is emerging for countries to perform case‐based surveillance of HIV programmes. Examples from the United Kingdom/Ireland, South Africa and Brazil are outlined below.

One of the first examples of national paediatric HIV surveillance was the National Study of HIV in Pregnancy and Childhood (NSHPC) in the United Kingdom and Ireland [42,43]. Since 1989, pregnancies in all HIV‐infected women and all new diagnoses of HIV in children have been reported to the NSHPC. These data are used to monitor the prevalence of diagnosed HIV infection in pregnant women and children, as well as track changes in the management of HIV in pregnancy and vertical transmission of HIV infection. Since 2000, the NSHPC has collaborated with the Collaborative HIV Paediatric Study (CHIPS), a multicentre cohort study that collects long‐term detailed HIV care and treatment information [44,45]. Standardized information is collected annually from treatment sites including virtually all HIV‐infected children and adolescents receiving HIV‐related care in the United Kingdom and Ireland. Through this, CHIPS is able to record HIV‐related immunological, virological and clinical outcomes during adolescence [46,]. The planned “CHIPS+” study will continue following all CHIPS patients after transition to adult care.

Another example of case‐based surveillance information captured at the point of care is the South African 3‐Tier ART Monitoring and Evaluation System [47,48]. The 3‐Tier System captures HIV‐specific patient level data at individual healthcare facilities according to one of three possible tiers (see Box 1). In three of the nine South African provinces, patient‐level data is sent from facilities through to provincial level, allowing for provincial level analysis of adolescent‐specific indicators. In the remaining provinces however data is exported into the aggregated District Health Information System (DHIS) software at the district level with loss of the ability to analyze patient‐level information by user‐specified age brackets. This is an example of where minor changes to the existing monitoring system's standard operating procedures could facilitate enhanced national level surveillance relevant to HIV‐infected adolescents. Mode of infection is not specifically captured, but with individual level data on age at diagnosis or age at first visit, a proxy indicator age less than a particular threshold, for example, 10 years at diagnosis or first visit, could be used to pragmatically differentiate the majority of vertically and horizontally HIV‐infected adolescents [27]. The 3‐Tier System does not capture indicators related to adolescent healthcare transition or transition to adulthood, but this may be feasible once such indicators are defined.

South Africa is also establishing a national cohort through the National Health Laboratory Service (NHLS) electronic database. Since 2004, the NHLS has been the primary provider of public sector laboratory testing in South Africa, including CD4 and HIV viral load tests. Through sophisticated record linkage techniques patients are either directly or probabilistically linked to test records that can be used to identify points in the cascade of HIV care. For example, the earliest recorded CD4 count for a patient would indicate entry into HIV care and the first recorded HIV viral load would indicate initiation of ART, as viral load testing is only conducted at or following initiation of ART and not prior to initiation, according to South African National HIV treatment guidelines [49]. This approach allows for tracking of patients even with migration or transfer of care between facilities and has the potential to identify healthcare transition between paediatric and adult HIV services. It does not allow for identification of deaths or additional clinical information. An adolescent cohort has been identified within the national NHLS cohort and has already provided useful estimates of the number of adolescents on ART and the proportion virologically suppressed [6]. Identification of presumed vertical HIV infection is possible when diagnosis is made during infancy with an HIV‐polymerase chain reaction test. It will be challenging to differentiate vertical from horizontal infection for children diagnosed with HIV late in childhood, and as with other systems, using a proxy of vertical transmission such as age at presentation of less than 10 years would need to be employed.

In Brazil, epidemiologic surveillance for HIV is conducted through the integration of national electronic information systems that are not necessarily specific to HIV. These include the notifiable disease information system, mortality information system, laboratory tests control system for CD4 and virologic data and the medication logistics control system for ART data [50]. Annual Brazilian HIV epidemic surveillance reports age disaggregate important indicators for 10–14‐year olds and 15–19‐year olds. In addition, the surveillance system is able to record mode of transmission to differentiate vertically from horizontally HIV‐infected adolescents [39]. Currently, the Brazilian surveillance system does not capture indicators related to adolescent healthcare transition or transition to adulthood; however, this is being discussed with the National AIDS Program.

While these case‐based surveillance systems offer tremendous potential they require consistent and complete reporting including from key populations often hesitant to be in contact with official health systems. Using case‐based surveillance for monitoring the outcomes of adolescents must be done with a comprehensive understanding of the limitations of the system.

**Table:** 

**Box 1. The 3‐Tier ART Monitoring and Evaluation System**
Implementation of the 3‐Tier System began in South Africa in 2010. Standardized HIV stationery is used for recording HIV‐infected patient related information including demographic, ART and laboratory information, at each care encounter. This information is captured into one of the tiers of the 3‐Tier System. The first tier comprises paper‐based registers. The second tier is a non‐networked electronic monitoring system that captures information identical to the paper‐based registers but with additional functionality to generate reports for programme monitoring and enhanced patient care and facility management. The third tier is a fully networked online patient management system that produces the same data as tiers one and two but with further functionality for real‐time co‐ordinated patient care across facilities. Data flows according to a standardized schedule from the facility level up to districts, provinces and finally to the National Monitoring and Evaluation Directorate. The 3‐Tier System has been implemented in 96% of healthcare facilities in South Africa with 91% on tier 2 or tier 3 and 90% having completed back capture of patient data to 2004. Records of approximately 3.5 million HIV‐infected South Africans currently in care and on ART are captured within the system. The 3‐Tier System is being expanded with implementation projects in Zimbabwe, Mozambique, Malawi, Guinea and Democratic Republic of Congo.

## WHO ART guideline revisions – the need for adolescent‐specific evidence

The 2016 revision of the WHO consolidated guidelines on The Use of Antiretroviral Drugs for Treating and Preventing HIV Infection, includes three adolescent specific recommendations addressing when to start ART, what antiretroviral regimens to use and guidance on the delivery of adolescent friendly health services (see Box 2) [22]. This comes out of increasing advocacy by adolescents themselves and recognition by service providers and programmes of the developmental differences between adolescents, children and adults and how these differences impact on the adolescent HIV disease course, treatment requirements and engagement with HIV services [3]. The strength of the recommendations made in the recent guideline revision is affected by a lack of specific evidence on optimal drug regimens and service delivery approaches for adolescents [51]. The process of recognizing that adolescents may require different recommendations to adults or children has highlighted the numerous gaps in the evidence for what the most appropriate and effective ART regimens are for this age group.

The additional effort required to fulfil ethical requirements related to research on people younger than 18 years of age can be a deterrent to their inclusion in pharmaceutical and other studies [52]. Even when adolescents are included, no or inappropriate age disaggregation of results e.g. stratifying by above and below 12 or 18 years of age, forfeits the opportunity to better understand adolescent drug metabolism, side‐effects and what the most effective ART regimen might be for adolescents living with HIV for example [53]. These research gaps impact critically on HIV programming for adolescents particularly related to procuring drugs and ensuring that adolescents receive the most effective ART regimens for long‐term health and wellbeing.

Increasing numbers of adolescents living with HIV will be starting ART and requiring adolescent‐appropriate ART services [54]. There is little HIV‐specific or general adolescent evidence however to inform the most effective service delivery interventions and approaches for this population [55]. Adolescents living with HIV are an extremely diverse group as presented in the article by Lam et al. in this issue. Despite their diversity, they experience common challenges particularly related to adherence, retention in care, sexual and reproductive health and mental health [56]. Research efforts require a focus on how best to provide sustainable and scalable services to this age group at all levels of the health system and particularly for the majority of adolescents living with HIV receiving their care in primary healthcare settings in low‐ and middle‐income countries.

Until recently, global and national programmatic reporting has traditionally disaggregated HIV data above and below 15 years of age. The recent WHO Consolidated Strategic Information Guidelines strongly recommend age disaggregation in 5‐year age bands, and with expanding electronic monitoring systems this is becoming feasible [54]. Where paper‐based reporting predominates though, the recommendation is to disaggregate into a few standard age groups, and the recommendation here for disaggregation of 5–14‐year olds as a single group hampers optimal tracking of younger adolescent outcomes [54]. To meaningfully impact on the outcomes and experiences of adolescents living with HIV and successfully provide services that meet their needs, means need to be found of measuring in both surveillance and research not only if adolescents are alive and on ART, but if they are thriving. This requires looking beyond routine indicators for programme monitoring, to additional indicators related to progression through adolescent‐appropriate developmental tasks and successful transition to adulthood.

### Conclusions and recommendations

The population of adolescents aged 10–19 years living with HIV continues to grow as survival of vertically HIV‐infected children and adolescents improves and new HIV infections, although declining, are still substantial [4]. Alongside this, mortality in older HIV‐infected adolescents continues to rise while estimates suggest that mortality in younger adolescents is starting to decline [4]. Reorientation of healthcare services and health information systems is needed to monitor the HIV epidemic in this diverse and complex population. Capacity for nationwide case‐based surveillance is growing; however, if these systems do not disaggregate data to determine outcomes in younger and older adolescents specifically, the opportunity for population level data in place of modelled estimates in this age group will be lost [47,50]. Without the ability of routine monitoring and surveillance systems to capture and disaggregate data by mode of transmission, the long‐term outcomes of vertically HIV‐infected children growing into adolescence and adulthood cannot be determined by these systems.

To facilitate HIV programming that is responsive to the vulnerabilities of this transitioning age group and ensure safe transitioning to adulthood for all adolescents living with HIV we recommend (Table 2):

**Table 2:** Recommendations to improve understanding of the adolescent HIV epidemic

Recommendation	Action level	Priority level
Disaggregation of routine monitoring, surveillance and research data by younger (age 10–14 years) and older (age 15–19 years) adolescents	National reporting Research Cohorts	High
Disaggregation of routine monitoring, surveillance and research data by mode of HIV transmission in older adolescents (age 15–19 years) and young adults (age 20–24 years)	Sentinel sites within routine monitoring systems Research cohorts	Medium
Expansion of case‐based national HIV surveillance particularly in high HIV burden countries	National Ministries of Health supported by non‐governmental partners	Medium
Streamlining consent and assent processes for inclusion of children and adolescents in research and population‐based surveys	Research ethics boards Regulatory bodies	High
Consensus on definitions related to adolescent transition to adulthood and adolescent healthcare transition, and integration of their measurement into routine national and global HIV programme reporting systems	Researchers National Ministries of Health Global reporting agencies Policy makers	Medium

1. Disaggregation of routine monitoring, surveillance and research data by age (younger adolescents, older adolescents, young adults) and where feasible by likely mode of HIV transmission, especially in older adolescents and young adults. This will aid identification and understanding of differences in trends in key outcomes including new infections, proportion on ART, virologic suppression, retention in care, mortality and differing service delivery needs in younger and older as well as vertically and horizontally HIV‐infected adolescents;
2. Continued expansion of case‐based national HIV surveillance particularly in high HIV burden countries, through the development and implementation of appropriate systems and tools. This will generate reliable population level data on the HIV epidemic as a whole and particularly for lower prevalence age groups including children and adolescents where modelled estimates lack precision;
3. Streamlining of processes to receive consent and assent for inclusion of children and adolescents in all forms of research including national population‐based surveys and pharmaceutical studies;
4. For indicators of adolescent transition to be included in and reported on by national HIV programs, consensus is needed across settings on how to define and measure successful transition through adolescence to adulthood and successful healthcare transition where applicable.

With advances in the efficacy of and access to ART regimens, this growing generation of adolescents living with HIV, both vertically and horizontally acquired, have the opportunity unlike generations before, to meaningfully contribute to society and thrive into adulthood. To fulfil this potential, a new paradigm of health system programming and monitoring is required, with the central involvement of adolescents in determining this trajectory.

**Table:** 

**Box 2. Summary of adolescent‐specific 2016 WHO recommendations**	
1. When to start ART in adolescents:	ART should be initiated in all adolescents living with HIV regardless of WHO clinical stage and at any CD4 count. *[conditional recommendation, low‐quality evidence]* As a priority ART should be initiated in all adolescents with severe or advanced HIV clinical disease (WHO clinical stage 3 or 4) and adolescents with a CD4 count ≤ 350 cells/mm^3^. *[strong recommendation, moderate‐quality evidence]*
2. First‐line ART for adolescents:	First‐line ART for adolescents should consist of two nucleoside reverse transcriptase inhibitors and a non‐nucleoside reverse transcriptase inhibitor or an integrase inhibitor. *[strong recommendation, low‐quality evidence]* TDF + 3TC (or FTC) + EFV as a fixed‐dose combination is recommended as the preferred option to initiate ART *[strong recommendation, low‐quality evidence]* TDF + 3TC (or FTC) + DTG or TDF + 3TC (or FTC) + EFV_400_ may be used as alternative options to initiate ART *[conditional recommendation, low quality evidence]*
3. Adolescent‐friendly health services:	Adolescent‐friendly health services should be implemented in HIV services to ensure engagement and improved outcomes *[strong recommendation, low‐quality evidence]*

## Author's contributions

ALS and MD conceived of the review and conducted background research. MM provided additional UNAIDS data and contributed to analysis and interpretation of the data. AA contributed intellectual content. ALS drafted the manuscript and all authors critically reviewed and approved of the manuscript.

## Acknowledgements

We would like to thank the following people for their guidance related to national surveillance and monitoring programs: Ali Judd (UK), Mhairi Maskew (South Africa), Meg Osler (South Africa), Jorge Pinto (Brazil), Gayle Sherman (South Africa), Lyson Tenthani (Malawi), Claire Thorne (UK), Hannock Tweya (Malawi), Rachel Vreeman (Kenya).
